# Rapid, single-tube method for quantitative preparation and analysis of RNA and DNA in samples as small as one cell

**DOI:** 10.1186/1472-6750-5-2

**Published:** 2005-01-13

**Authors:** Cristina Hartshorn, Aleksandra Anshelevich, Lawrence J Wangh

**Affiliations:** 1 Department of Biology, Brandeis University, Waltham MA 02454-9110, USA; 2 Current address: Division of Cardiology, Beth Israel Medical Center, Harvard Institutes of Medicine, Boston, MA 02115, USA

## Abstract

**Background:**

Current methods for accurate quantification of nucleic acids typically begin with a template preparation step in which DNA and/or RNA are freed of bound proteins and are then purified. Isolation of RNA is particularly challenging because this molecule is sensitive to elevated temperatures and is degraded by RNases, which therefore have to be immediately inactivated upon cell lysis. Many protocols for nucleic acids purification, reverse transcription of RNA and/or amplification of DNA require repeated transfers from tube to tube and other manipulations during which materials may be lost.

**Results:**

This paper introduces a novel and highly reliable single-tube method for rapid cell lysis, followed by quantitative preparation and analysis of both RNA and/or DNA molecules in small samples. In contrast to previous approaches, this procedure allows all steps to be carried out by sequential dilution in a single tube, without chemical extraction or binding to a matrix. We demonstrate the utility of this method by quantification of four genes, *Xist*, *Sry *and the two heat-inducible *hsp70i *(*hsp70.1 *and *hsp70.3*), as well as their RNA transcripts in single mouse embryos and in isolated blastomeres.

**Conclusion:**

This method virtually eliminates losses of nucleic acids and is sensitive and accurate down to single molecules.

## Background

Real-time polymerase chain reaction (PCR) in combination with reverse transcription (RT) provides a powerful tool for accurate quantification of DNA and RNA copy numbers and has opened the way to the study of subtle modulations of gene expression in small numbers of cells, as well as small-scale genetic analyses aimed at establishing chromosome numbers, the presence of mutations, or allele dropout. The reliability of these measurements, however, depends on the accuracy of each step, including preparation and recovery of RNA and/or DNA, reverse transcription of RNA into cDNA, and quantifiable and specific amplification of all desired sequences. The importance of optimizing each of these steps is well recognized [1], as is the need to minimize the number of tube-to-tube transfers in order to avoid the loss of templates and decrease the risk of contamination. This risk is posed by environmental RNases, material carried over from sample to sample, as well as previously generated amplicons present on laboratory equipment. Sequential performance of several steps in a single-tube is therefore highly desirable, especially when starting with small numbers of target molecules, such as chromosomes of individual cells or a few virus particles [2-5].

Our laboratory has already demonstrated that bound proteins prevent reliable PCR amplification of genomic DNA and that a thorough proteolytic digestion followed by heat inactivation solves this problem [6]. For accurate gene expression studies, RNA molecules also need to be released intact and free of proteins from all subcellular compartments, but proteases cannot be used both because they are not fast enough to inhibit the RNases (particularly endogenous RNases, released in the sample upon cell disruption) and because RNA is sensitive to the high temperatures required for protease inactivation [7]. Commercial kits for RNA purification therefore commonly employ either chaotropic agents or lysis buffers containing strong detergents, or a combination of the two, in order to achieve rapid denaturation of proteins. Nucleic acids are then extracted to remove these chemicals, because their presence interferes with subsequent enzymatic reactions. Alternatively, some RT-PCR kits bypass nucleic acid purification in favor of a simple dilution step, but in this case only a small aliquot of the lysed sample can be added to the RT mixture, due to volume restrictions. This approach introduces imprecision of its own and makes single cell analysis impossible. On the other hand, gentler lysis conditions that are compatible with single-tube analysis of a whole small sample do not remove proteins completely, resulting in substandard template preparations. For instance, protocols involving simple freeze-thaw cycles to produce cell lysis do not generate protein-free RNA or DNA. Similarly, mild detergents that do not lyse the nuclear membrane preclude quantification of DNA or RNA located in the nucleus, and are unlikely to completely remove proteins bound to cytoplasmic RNA.

The chaotropic agent guanidine isothiocyanate (GITC) has long been the chemical of choice for nucleic acid preparation. It is particularly useful for RNA studies [8,9], because it rapidly denatures all cellular proteins, as well as serum proteins, including RNases, added to culture media. GITC has also proven superior to all other tested methods for the recovery of either DNA or RNA extracted from mummified tissue [10]. Due to its strong chemical action, GITC at high concentrations offers the further advantage of allowing safe storage of the samples until they are processed for quantification. For the same reason, however, all traditional protocols require removal of GITC prior to RT and PCR to avoid inactivation of the enzymes involved. Typically GITC is removed by extraction with phenol-chloroform and purification of the nucleic acids through alcohol precipitation cycles [9], or by absorption of the freed RNA to a matrix such as glass fiber filters, silica-gel membranes, magnetic beads or proprietary compositions, usually followed by elution in a relatively large volume. Both these approaches are time-consuming and involve a number of steps that can lead to incomplete RNA recovery.

In view of these limitations we devised an alternative strategy in which the sample is collected and denatured in a minimal volume of a GITC solution, briefly heated to allow dry storage, and, when needed, is directly analyzed in the same tube by performing quantitative RT-PCR in a volume large enough to lower GITC concentration to negligible levels. This new procedure, hereafter referred to as PurAmp (patent pending) and described here in full for the first time, is the only available method that allows a whole sample, such as a single embryo or cell, to be processed from lysis to RT-PCR in the same tube, under conditions that permit precise quantification of both RNA and genomic DNA copy numbers. This fully optimized method is sensitive enough to detect specific sequences within a single chromosome in one cell, yet robust enough to measure the presence of thousands of RNA molecules released from hundreds of cells.

PurAmp has made it possible for us to conveniently investigate expression levels of two gene types essential for early mouse embryo development: *Xist*, responsible for X-inactivation and dosage compensation in female cells [11-13], and the heat-shock inducible *hsp70.1 *and *hsp70.3*, jointly called *hsp70i *[14,15]. *Xist *RNA is a noncoding transcript that exerts its particular function of gene-silencer by coating the inactive X-chromosome. Unlike *hsp70i *RNA and most other mRNAs, it is therefore localized in the cell nucleus and particularly challenging to extract. Besides their biological relevance, both *Xist *and the *hsp70i *genes offer the advantage of naturally-occurring unambiguous controls for the specificity of transcripts amplified with RT-PCR. *Xist *RNA is, in fact, virtually absent from male cleavage stage embryos [16-19], while *hsp70i *RNA is predominantly synthesized in response to stress although minimal levels of *hsp70i *transcripts are normally present in embryonic cells. A careful quantitative analysis of *hsp70i *heat and culture stress-response in preimplantation embryos at different developmental stages, and its implications for development, will be presented elsewhere (C. Hartshorn, A. Anshelevich and L. J. Wangh, in preparation). In addition, we have been able to detect and quantify the genomic sequences of *Xist*, the *hsp70i *and the male sex-determining gene *Sry *[20]. Because the number of these sequences is known and very low in samples comprised by an identifiable number of cells, such as early embryos that have undergone few cleavages, their precise quantification provided an optimal internal control to demonstrate the strength of this novel technique. The quick and reliable detection of DNA (or RNA) in very low copy number is, however, not limited to the role of internal control, but holds much wider utility for a variety of applications such as genetic studies and detection of viral sequences in a sample. Further, this method can be successfully employed for the study of individual cells, as shown by the present report, and is easily adaptable to analysis of subcellular fractions or aliquots from bodily fluids; it also minimizes the use of toxic chemicals and the possibility of contamination, while allowing dry storage of the collected samples. All these features concur to render PurAmp ideally suitable for fast but highly sensitive gene and gene expression screening of multiple samples, including small whole specimens or fractions of larger ones.

## Results

### Single-tube *Xist *RNA, *Xist *DNA and *Sry *DNA quantification in individual male and female blastocysts

The PurAmp method presented in this study is performed in a single tube from cell lysis to cDNA or genomic DNA amplification, thus eliminating possible loss of template molecules due to procedures such as phase separation and recovery, repeated washing and re-suspension of nucleic acid pellets, elution from binding matrices and vessel-to-vessel transfer. This strategy offers an immediate improvement in the precision of gene expression analyses, at the same time shortening considerably the experimental protocol compared to traditional methods.

In order to validate our method, we initially measured the *Xist *RNA content of a group of female mouse embryos at the blastocysts stage and quantified *Xist *and *Sry *DNA copy numbers in their male counterparts. Our previous analysis of these parameters in single female and male mouse embryos at different developmental stages provided us with an ample pool of data obtained with commercially available nucleic acids preparation methods [17-19], which we used as a reference for comparison with our new results.

**Figure 1:**
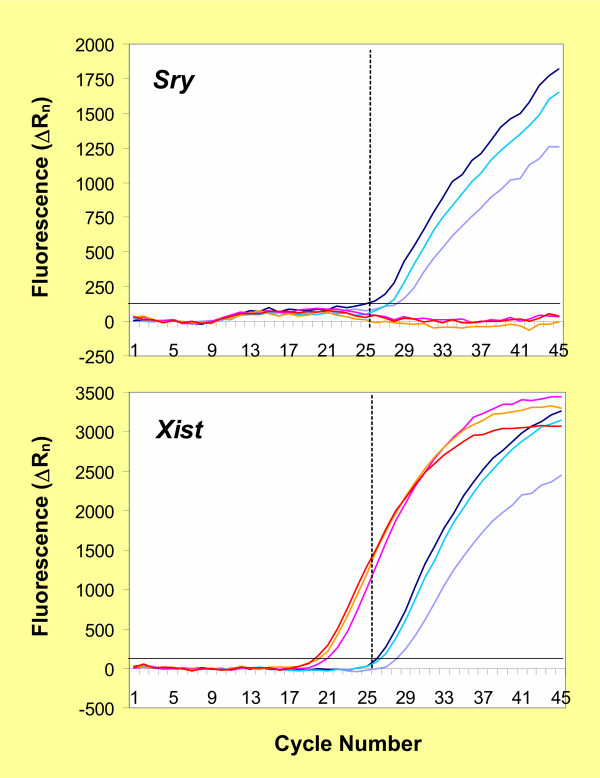
**Multiplex *Xist/Sry *template detection in individual blastocysts processed with the single-tube PurAmp method. ***Sry *(upper panel) and *Xist *(lower panel) real-time PCR plots generated by six single embryos at the blastocyst stage processed via PurAmp. All steps, starting with cell lysis, were performed by progressive dilution in the same optical-grade tube. *Xist *and *Sry *amplicons (genomic DNA plus cDNA when RNA was present) were detected simultaneously by using sequence-specific molecular beacons. Each color identifies a single embryo and is used in both panels. Female embryos (lines in red hues) were easily distinguished from male embryos (lines in blue hues) based on the presence of *Xist *RNA, which causes the female *Xist *signals to be much earlier than the male *Xist *signals, and on the absence of the Y-chromosome-specific *Sry *gene. Quantification of *Xist *and *Sry *copy numbers (given in the text) was obtained based on the C_T _of the real-time PCR plots (see Methods), using a genomic DNA standard curve as detailed in Fig. 2. The horizontal line in each chart indicates the threshold used to determine the C_T _values. The dashed vertical lines facilitate the comparison of *Sry *and *Xist *C_T _values in male embryos.

Figure 1 shows the real-time PCR plots obtained from six PurAmp-treated single embryos. Following RT, the accumulation of multiplexed *Xist/Sry *amplicons was detected using two molecular beacons conjugated to different fluorescent dyes. Both amplicons span intronless sequences of the genes [18], thus allowing in either case the simultaneous measurement of cDNA (when present) and genomic DNA copies. Each color in the plots of Fig. 1 identifies a specific embryo and is used for both its *Sry *and *Xist *signal. Three embryos were identified as male based on the presence of the *Sry *amplicon (Fig. 1, upper panel, lines in blue hues) and on the fact that in each case the *Xist *fluorescent signal (Fig. 1, lower panel, lines in blue hues) arose at the same "threshold cycle" (C_T_) as the *Sry *signal (see Methods for a definition of C_T _and details on signal quantification). This indicates the presence of the same number of copies of *Sry *and *Xist *templates, as expected for male blastomeres that contain one copy of the *Sry *gene on the Y-chromosome and one copy of the *Xist *gene on the X-chromosome. Neither gene is expressed in male blastocysts [16,17], and therefore these three samples contained only genomic *Xist *and *Sry *DNA. The three remaining embryos did not generate any *Sry *signal (Fig. 1, upper panel, lines in red hues), while their *Xist *signals arose significantly earlier than the others (Fig. 1, lower panel, compare lines in red and blue hues). Based on these data they could be identified as female embryos which contained thousands of copies of *Xist *RNA in addition to two copies of genomic *Xist *DNA per cell. These results are fully in agreement with those of our earlier analyses using traditional methods of nucleic acid preparation.

**Figure 2:**
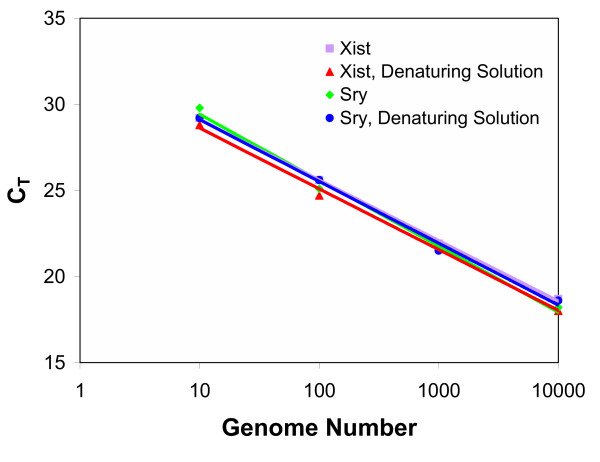
***Xist/Sry *real-time PCR efficiency in the presence or absence of diluted denaturant. **Standard curve used for the quantification of multiplexed *Xist *and *Sry *amplicons, obtained by serial dilution of male mouse genomic DNA at known copy number (abscissa). The *Xist *(light purple) and *Sry *(green) signals had very similar C_T _values (ordinate) at every DNA concentration tested, because the two PCR reactions had been optimized to be equally efficient. The addition of denaturing solution at the concentration used in the PurAmp assays did not inhibit PCR (red, *Xist*; blue, *Sry*), as also seen in the case of the *hsp70i *amplicon.

Quantification of the real-time PCR data obtained from the three male embryos in Fig. 1 indicated that, on average, each embryo contained 125 ± 83 (mean ± s.d.) copies of *Xist *genomic DNA, consistent with the previous estimate of 165 ± 101 genomes per male blastocyst [17]. Both values are higher than the expected cell number per embryo at this stage (60–100, depending on culture conditions), due to endoreduplication in trophoblast cells [21], a phenomenon also responsible for some variability between samples. In contrast, a total of five female blastocysts analyzed via the PurAmp method yielded an average of 12,600 ± 5079 copies of *Xist *cDNA + genomic DNA per embryo. Since each female blastocyst contains about 250 copies of the *Xist *genomic sequence (twice the number of a male embryo), the accumulation of *Xist *RNA per female blastocyst averages above 12,000 copies, considerably higher than our previous measurement of 6797 ± 2894 copies obtained using a multistep nucleic acids isolation procedure [17]. The data in Figure 2 demonstrate that the amplification efficiency of both the *Xist *and *Sry *sequences is neither decreased nor increased by the presence of diluted denaturing solution (0.4 mM GITC, as in the PurAmp protocol) during real-time PCR. In fact, additional experiments revealed that *Xist/Sry *real-time PCR was unaffected by a GITC concentration as high as 20 mM (not shown). Taken together these results suggest that the higher levels of *Xist *cDNA measured using the PurAmp method are due to an improvement in RNA recovery at the initial step of cell lysis.

### Quantification of low-to-high *Xist *RNA and DNA copy numbers in single embryos and blastomeres

**Figure 3:**
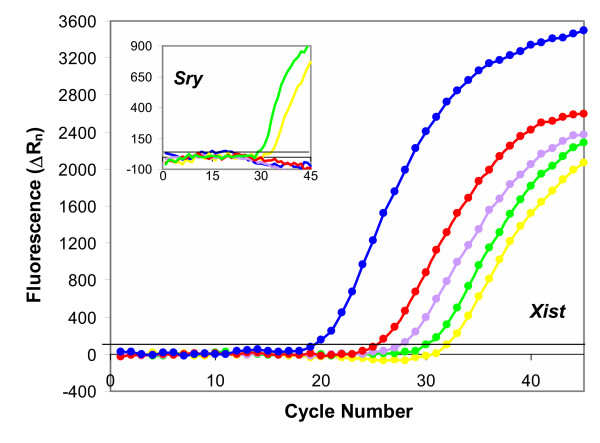
**Accuracy and efficiency of the PurAmp protocol for wide-range copy number quantification. ***Xist *real-time PCR plots generated from individual embryos or cells processed with the PurAmp method. In the samples analyzed, the number of *Xist *genomic DNA + cDNA (RNA) templates increased right-to-left from 3 (yellow) to 10,000 (blue), as detailed in the Results section. Yellow, 3-cell male embryo; green, 4-cell male embryo; light purple, single blastomere from a 4-cell female embryo; red, 4-cell female embryo; blue, female blastocyst. Male samples also produced an *Sry *signal (inset), confirming sex assignment based on the presence or absence of *Xist *expression.

As shown above, blastocysts are comprised by many cells and contain hundreds of copies of the *Xist *and *Sry *genes and thousands of copies of *Xist *transcripts, with rather wide sample-to-sample fluctuations. In order to more carefully determine the quantitative capability of the new assay, we next analyzed embryos at earlier developmental stages containing lower and, in some cases, precisely known numbers of template copies. Figure 3 illustrates the real-time PCR plots of the *Xist *amplicons generated in the course of two separate experiments by, right-to-left, i) a 3-cell male embryo (yellow); ii) a 4-cell male embryo (green); iii) a single blastomere isolated from a 4-cell female embryo (light purple); iiii) a 4-cell female embryo (red); iiiii) a female blastocyst (blue). The gender of each embryo was confirmed by the detection of an *Sry*-specific fluorescent signal in male samples (Fig. 3, inset). The quantitative analysis of these results confirmed that the 3-cell male embryo contained 3 copies of the *Xist *gene, while the 4-cell male embryo contained 6 copies of the *Xist *gene, indicating that DNA duplication had occurred in two of the blastomeres. It has long been known that two of the blastomeres of a 4-cell embryo divide ahead of the other two [22], an observation in agreement with our finding. The numbers of *Xist *templates measured in these male embryos also confirmed the expectation that these samples did not contain *Xist *RNA because *Xist *is not expressed in male cells [13,17]. Conversely, the *Xist *signal of the female 4-cell embryo arose about five cycles earlier than the *Xist *signal of the 4-cell male embryo (compare red and green curves), denoting the presence of *Xist *transcripts (157 copies of *Xist *RNA assuming diploidy of all cells and calculated from a total of 165 cDNA + genomic DNA templates), albeit at considerably lower levels than those measured in the female blastocyst (9750 copies of *Xist *RNA assuming the aforementioned average number of genomes per blastocyst of 125, and based on 10,000 copies of total *Xist *cDNA + genomic DNA templates). These measurements are consistent with other studies demonstrating that *Xist *transcripts are accumulated in the developing embryo beginning at the late 2-cell stage [23] and with our previously published *Xist *developmental profile [17].

**Figure 4:**
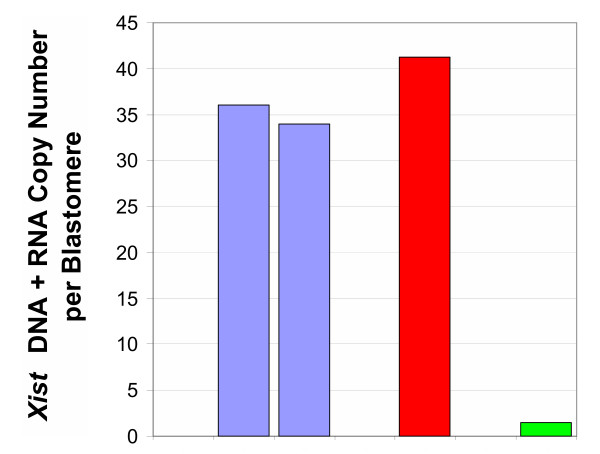
**Quantification of *Xist *RNA levels in isolated blastomeres and whole embryos at the 4-cell stage. **Comparison of *Xist *genomic DNA + cDNA (RNA) levels measured in single blastomeres isolated from a 4-cell female embryo and in whole 4-cell embryos of either sex. Whole embryo data are presented on a per cell basis. Light purple bars, two individual blastomeres harvested from the same female embryo. Red bar, whole female embryo (per blastomere average). Two *Xist *template copies in each female cell are accounted for by the presence of *Xist *genomic DNA, while the remaining copies signal the presence of *Xist *RNA. Green bar, whole male embryo (per blastomere average): as expected, the number of *Xist *copies in this sample corresponds to the presence of one copy of the *Xist *gene per cell and the absence of *Xist *transcripts. Sexing was confirmed in all samples by the presence or absence of the *Sry *gene.

The quantitative accuracy of the PurAmp method was further confirmed by the fact that the *Xist *signal generated by a single blastomere isolated from a 4-cell female embryo arose 2.2 cycles later than the *Xist *signal of the whole 4-cell female embryo (compare light purple and red curves, Fig. 3). A left-to-right shift of 2 cycles is exactly what is expected for a fourfold decrease in template numbers quantified by real-time PCR amplification. Figure 4 illustrates this point by showing *Xist *RNA + genomic DNA levels in two individual blastomeres isolated from a 4-cell female embryo, as compared to *Xist *template levels measured in intact 4-cell embryos of different sex and then calculated on a per cell basis. Even at this early developmental stage, the presence of *Xist *RNA is clearly detectable in the female samples, absent from the male, and not affected by the blastomere isolation procedure [18].

### *Hsp70i *RNA and DNA measurements in heat shocked and non-heat shocked single embryos and blastomeres

In order to more extensively test the validity of the PurAmp approach to template quantification in single cells, we measured transcript levels of the heat shock-inducible genes *hsp70.1 *and *hsp70.3 *in blastomeres isolated from embryos at the pre-compaction 8-cell stage, when cells can be easily counted and separated. The sequences of these two genes are almost entirely identical, they are located on the same chromosome and they encode the same protein [14,15]. For this reason, there has been some confusion in their identification and nomenclature in past studies. Heat-inducible *hsp70 *transcription, previously indicated as *hsp70.1 *expression, is now more precisely designated as the sum of *hsp70.1 *and *hsp70.3 *(*hsp70i*) RNAs.

A preliminary set of experiments was carried out on embryos at the blastocyst stage, when heat shock response is fully established [14], with the goal of evaluating the effect of hyperthermia on *hsp70i *expression. Like *Sry*, the *hsp70i *are naturally intronless genes and therefore once again our pair of PCR primers simultaneously amplified both genomic DNA and cDNA sequences. The data in Table 1 clearly indicated that heat shock (see Methods) produced a sharp rise in *hsp70i *template numbers due to the presence of thousands of copies of *hsp70i *RNA, although these numbers were considerably lower when samples were prepared with a multistep/multitube phenol-chloroform extraction [17,18] rather than with the PurAmp method. During these initial experiments, embryos were allowed to recover for 30–40 minutes after heat shock. Under these conditions, however, only five out of seven blastocysts exposed to hyperthermia showed an increase in *hsp70i *RNA levels. Based on these results, the duration of the recovery period was increased to at least two hours in all following experiments, eliminating the finding of "non responsive" embryos.

Copy numbers of *hsp70i *RNA were then quantified in whole 8-cell embryos that had or had not been exposed to a temperature increase, as summarized in Table 2. The number of *hsp70i *genomic DNA copies measured in the absence of RT was consistent with the presence of four copies of the genes per cell, one *hsp70.1 *and one *hsp70.3 *on each chromosome 17, and with the fact that some of the cells analyzed had already duplicated their DNA. Only a minimal amount of *hsp70i *RNA, calculated as the difference of template copy numbers obtained with and without reverse transcription (*hsp70i *cDNA + genomic DNA less *hsp70i *genomic DNA), was present in non-heated embryos, indicating that the embryos were not stressed by culture conditions [24].

Expression of *hsp70i *RNA increased sharply after a 30-minute heat treatment, followed by a recovery period of either 2 or 3 hours necessary for transcripts synthesis and accumulation. Some of the heat-shocked embryos were harvested intact, while others were dissociated in single blastomeres. The average numbers of *hsp70i *template copies per blastomere were calculated from the isolated cells (not all cells of dissected 8-cell embryos could be recovered) and compared to the average per blastomere values obtained from whole embryos. The results show that the amount of *hsp70i *RNA + genomic DNA per cell calculated by these two approaches is very similar. A post-heating recovery period of 3 hours rather than 2 hours increased the *hsp70i *RNA levels only slightly, indicating that the onset of transcription and the major build-up in RNA occur quickly. Single cells derived from non-heated embryos contained only trace amounts of *hsp70i *RNA, consistent with whole embryo measurements, and PCR efficiency was, again, unaffected by the PurAmp components (not shown).

### Efficiency of DNase treatment within the PurAmp protocol

**Figure 5:**
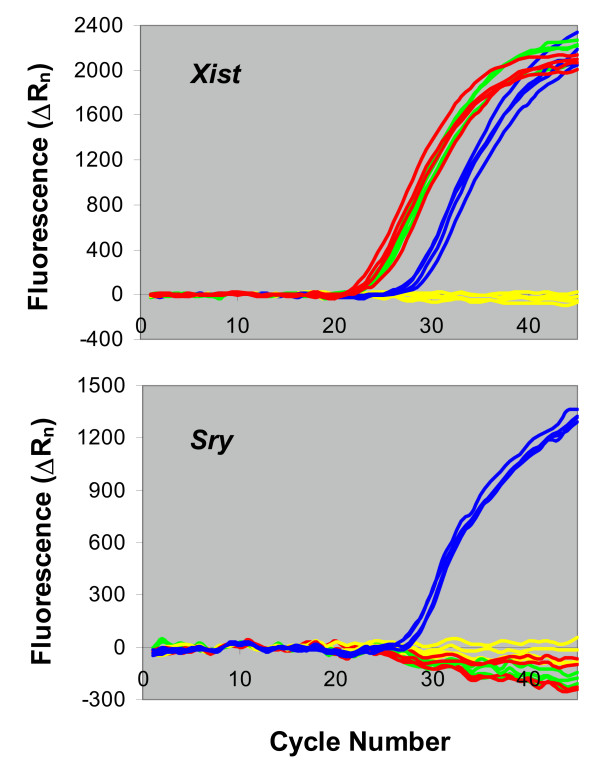
**Introduction of a DNase step within the single-tube PurAmp protocol for *Xist/Sry *quantification. **Effect of DNase digestion on *Xist *(upper panel) and *Sry *(lower panel) template copy numbers measured in male and female single embryos at the morula stage. Male embryos: blue, non-treated; yellow, DNase-treated. Female embryos: red, non-treated; green, DNase-treated. The DNase enzyme displayed full activity under the conditions tested, as shown by the absence of genomic DNA in treated male samples and detailed in the text.

The PurAmp method described above automatically results in the quantitative recovery of genomic DNA, which can then be used as a quality control and a convenient internal standard for the simultaneous recovery and measurement of mRNA [[17-19], see Discussion]. Applications such as microarray analysis, however, are based on RNA-only amplification. For this reason, we introduced a DNase digestion step preceding RT in the *Xist/Sry *PurAmp protocol and analyzed the efficacy with which the genomic DNA was degraded. Genome numbers in embryos at the morula stage were calculated by counting *Xist *and *Sry *copies in five male samples (as detailed for blastocysts, see above) and averaged at 21.3 ± 8.9, consistent with the fact that embryos at this stage are normally comprised of 16-to-32 cells. After treatment with DNase I, only 0.8 ± 1.5 genomes per embryo were still present in a group of 15 male samples, demonstrating that the enzyme had successfully degraded 96.3% of the DNA. Figure 5 illustrates the effects of DNase digestion on *Xist *(upper panel) and *Sry *(lower panel) DNA in a group of eight male and eight female single embryos. As expected, control male samples (blue lines) contained equal numbers of *Xist *and *Sry *copies, as shown by the equal C_T _values, corresponding to the genomic DNA copy number. Both amplicons were absent from DNase-treated embryos that could be identified as male because they were devoid of *Xist *RNA (yellow lines). In contrast, control female samples (red lines) contained *Xist *RNA and DNA but lacked *Sry*. DNase treatment caused a delay in the *Xist *signals arising from female embryos (green lines), consistent with the elimination of all 42 copies of genomic *Xist *DNA in each embryo plus some decrease in the number of *Xist *transcripts. The amount of RNA recovered in these samples averaged at 75% of control levels. The fact that some RNA is lost during the DNase step is not surprising as it is well known that some RNA hydrolysis is unavoidable (see Discussion), and is not linked to the single-tube procedure. We anticipate that further optimization of the available DNase protocols and reagents will minimize this problem. Thus, the data in Fig. 5 demonstrate overall that a DNase digestion step can be successfully inserted within the PurAmp procedure, without disruption of the DNase enzymatic activity.

## Discussion

It is increasingly clear that individual cells in a population do not exhibit identical patterns of gene expression and, hence, that expression profiling is more informative if it is quantitative and carried out at the single cell level [25-27]. This consideration is particularly relevant to current efforts aimed at understanding early mammalian embryogenesis in which totipotent cells generated during the first few cell divisions gradually become committed to particular lines of development. The mechanisms of this process are under intensive scrutiny and appear to be rooted in differential gene expression resulting from epigenetic modifications. It is in this context that we have been measuring RNA levels in single cells of cleaving embryos [18,19] and it is to increase the reliability of these measurements that we have now developed the PurAmp method. This completely single-tube approach is easy to use, eliminates loss of material, and improves the quantitative accuracy of gene and gene expression studies.

First, cell lysis and protein denaturation occur very rapidly upon delivery of the sample to crystalline GITC, thus ensuring both protein removal from DNA and RNA and inactivation of cellular nuclease that would otherwise quickly degrade RNA [8]. Transcripts localized in the nucleus, such as *Xist *RNA, are freed and made available to reverse transcription as well as cytoplasmic mRNA molecules, a result unattainable by mild detergent treatment that leaves nuclei intact [25]. Second, the brief heating period after cell lysis enhances complete denaturation of proteins and also reduces the volume of the sample, thereby further increasing the guanidine concentration. The semi-dry sample can then be safely stored without risk of nuclease activity. Third, carrying out cell lysis in nanoliter volumes allows a manifold dilution of the chaotropic agent after addition of the RT cocktail, so that RT can be performed on the whole sample and in the same vessel in which it was collected without inhibition of the enzymatic activity. Finally, RT and PCR can be carried out immediately after cell lysis, rather than after cumbersome and lengthy nucleic acid preparation procedures, thereby further reducing the time required to process many samples, as well as the risk of contamination.

**Figure 6:**
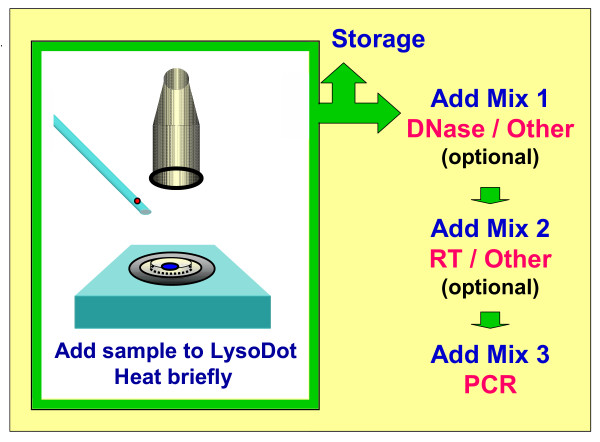
**Easiness and versatility of the PurAmp method. **In a streamlined version of the procedure, LysoDots (20 nl or less, see Methods for a definition) are dispensed to the lids of PCR tubes resting on a heating block. The LysoDots are quickly dried before adding the samples in a minimal volume of PBS. A second brief heating reduces the lysed samples to the semi-dry state, ready for storage or immediate processing, which may include a variable number and type of enzymatic reactions prior to the final PCR step. This will be a real-time PCR assay when quantification of the templates is desired.

Our quantitative measurements of *Xist *RNA levels in developing mouse embryos highlight one of several merits of the PurAmp method over the traditional, multistep approach to nucleic acids purification [17,18]. In fact, while genome numbers obtained with the two methods are similar as expected, *Xist *RNA levels are higher with the single-tube protocol. The same culture conditions and procedures were used in the two groups of experiments, making it unlikely that differences in embryo quality were the cause of the increase in *Xis*t RNA. We, therefore, conclude that the higher levels of *Xist *RNA observed using the new procedure reflect improved template preparation with efficient inactivation of RNases and reduced loss of RNA molecules. *Xist *RNA is known to trigger X-chromosome silencing through interactions with numerous proteins and possibly with the nuclear matrix scaffold [28,29]. The results presented in this study clearly show that the very high initial concentration of GITC thoroughly breaks up protein-RNA interactions, but the denaturant does not inhibit subsequent RT once is diluted. Similarly, our quantification of *hsp70i *templates in heat-shocked blastocysts supports the view that larger pools of RNA are detected in PurAmp-treated samples than when using phase separation-based nucleic acid extraction. The later method, in fact, presents several steps that require extreme care to avoid loss of material, including complete recovery of the upper phase, thorough precipitation of all nucleic acids molecules, and repeated re-suspension and washing of barely visible pellets. All these manipulations render the results obtained with this technique particularly operator-dependent, while, in contrast, PurAmp simply requires sequential addition of reagents into the same tube. Once the sample is delivered to the LysoDot in the reaction vessel (see Figure 6 and Methods section), therefore, this technique is much less dependent on the operator's specific skill.

Individual blastomeres of pre-compaction mouse embryos are easily harvested due to their size, and laser zona drilling efficiently preserves RNA pools allowing dependable single-cell analysis [18]. We thus used measurements of *Xist *and *hsp70i *RNA levels in single blastomeres to further validate the quantitative accuracy and reliability of the PurAmp method, as shown by the fact that transcript levels in individual cells are comparable to average RNA levels per cell calculated from whole embryos. Based on these results we anticipate that PurAmp will prove useful for quantification of RNA levels in small pieces of tissue from many sources, as well as single cells and even fractions of cells such as neuronal dendrites and axons [30]. The small volume in which denaturation is carried out is also amenable to analysis of biological material isolated by laser capture microdissection or laser pressure catapulting [31].

Genomic DNA has recently been proposed as the optimal standard for gene expression studies [32] and it is the required internal standard when cDNA is quantified with the strategy of amplification competition [33]. In this case, DNA and RNA are purified together, as in our experiments, and one set of primers is designed to co-amplify a genomic sequence that spans an intron as well as the corresponding intronless cDNA. The alternative strategy that we developed makes use of primer sets that do not span introns and therefore amplify genomic DNA sequences that have the same length and composition as their corresponding cDNA's, eliminating any possible difference in PCR efficiency for the two types of templates [[17-19]; C. Hartshorn, A. Anshelevich and L. J. Wangh, in preparation]. We have found it very informative to measure genomic DNA copy numbers in addition to RNA levels of the genes under study, because this strategy provides a reliable internal control for primer specificity and for nucleic acids recovery, particularly when performing single-cell analyses. In the case of early mouse embryos, detection of the *Sr*y gene, which is not expressed at those stages, has also allowed us to identify the sex of each embryo.

The recovery and quantification of genomic DNA together with RNA has previously enabled us to establish genome number averages for developing embryos [17,19]. While these numbers are very similar to the number of cells in early embryos, measurements of DNA copies are more accurate because they indicate whether the cells have completed S phase. Genome quantification becomes even more critical after the late 8-cell stage, because the embryos compact making it very difficult to count individual cells. Moreover, endoreduplication takes place in trophoblasts at the blastocyst stage, greatly increasing the number of genome copies present in those cells [21]. All these factors render the counting of DNA copy numbers important if gene expression data are to be calculated on a per-genome basis, independently from a cell's ploidy.

A further reason to preserve DNA molecules in preparations for RT-PCR is that all DNase digestion protocols currently available lead to partial hydrolysis of RNA when the enzyme is heat-inactivated in the presence of divalent cations at the end of the reaction [34]. We have consistently found a decrease in amplified cDNA in DNase-treated samples, particularly when performing RNA isolation with traditional methods (unpublished results), even when a chelating agent was added prior to the heating step. Incomplete RNA recovery after DNase inactivation in the presence of EDTA was not evident in past reports, due to the use of non-quantitative methods of nucleic acids analysis [35]. Our real-time PCR results, however, agree with numerous more recent findings [see ref. [36] for an overview of DNase-related problems]. Efforts have been made, therefore, to devise alternative ways to eliminate the DNase once digestion has occurred. These methods, however, depend on removal of the enzyme which, in turn, implies manipulations such as phenol extraction that may still generate nucleic acids loss.

For all of the above reasons as well as the fact that we are working with very small amounts of material, we prefer single-tube preparation-to-amplification of both RNA and DNA templates, an approach made possible for the first time by the procedure described in this paper. Previously reported single-tube template preparation protocols, in fact, have been aimed at measuring only specific RNAs and employ lysis buffers containing low concentrations of the mild detergent NP-40 [25,37], or they bypass the lysis step altogether and are limited to neuron studies [38]. While these methods are valuable for detection of protein-free RNA molecules, they utilize non-denaturing conditions, as clearly demonstrated by the addition of proteic RNase inhibitors to the extraction buffers, and therefore preclude quantitative analysis of DNA [6] as well as of protein-bound RNA pools.

## Conclusions

Due to its ability to thoroughly remove proteins from both RNA and DNA molecules in a rapid and simple way, PurAmp is suitable to a wide variety of applications, including gene expression quantification, studies on genetic mutations, and viral detection. Because the presence of DNA is undesirable for certain applications, such as microarray-based expression profiling, we have also shown that a DNase digestion step can be easily included in the single-tube format. We anticipate that treatment with other enzymes, such as cellulase in the case of plant cells, can similarly be inserted into the PurAmp protocol to digest other "undesired" components of particular cells prior to amplification. Thus, PurAmp is a very flexible technique that affords the investigator a variety of ways of processing the contents of a lysed sample with a heightened level of precision (Fig. 6).

## Methods

### Embryo culture and single blastomere isolation

For most experiments, frozen late 2-cell stage embryos (B6C3F1 females bred with B6D2F1 males) were obtained from Embryotech Laboratories, Inc. (Wilmington, MA), and were cultured as previously described [17] until the desired stage of development. For the DNase experiment, frozen 8-cell embryos obtained from the same source were grown to the morula stage. Blastocyst stage embryos used for *hsp70i *measurements were also grown from frozen 8-cell embryos.

Single blastomeres were isolated from either 4-cell embryos (for *Xist *measurements) or pre-compaction 8-cell embryos (for *hsp70i *measurements) after drilling the zona using a ZILOS-tk™ zona infrared laser optical system (beam = 1480 nm) (Hamilton Thorne Biosciences, Inc., Beverly, MA), according to a procedure developed in our laboratory and described elsewhere [18,19].

### PurAmp multiplex measurements of *Xist/Sry *RNA + DNA in individual embryos or blastomeres

All experimental procedures were carried out using rigorous precautions aimed at avoiding or destroying environmental RNases contamination [17-19].

Dried droplets of denaturing solution, hereafter called "LysoDots", were prepared prior to embryo collection by delivering 20-nl aliquots of the denaturing solution (see below) to the inside surface of the lids of PCR-grade reaction tubes (Applied Biosystems, Foster City, CA). Precise measurement of the droplets size was obtained following the method previously described by Wangh [39]. The denaturing solution composition was: 0.25% sarcosyl, 2 M GITC, 100 mM β-mercapto-ethanol, 0.01 M sodium citrate, pH 7.0 (all reagents from Stratagene, La Jolla, CA), 1% (vol/vol) dimethylsulfoxide (Sigma Chemical Company, St. Louis, MO). LysoDots were prepared in advance, allowed to dry under sterile conditions, and then stored at room temperature in closed PCR tubes.

Immediately before harvesting, individual embryos were placed in 3 ml of Dulbecco's PBS devoid of calcium and magnesium chloride [17]. Dulbecco's PBS containing 0.4% polyvinyl pyrrolidone (both products from Sigma) was used when isolating single blastomeres [18]. After one wash in the same buffer, each embryo or cell was aspirated into a glass capillary having an internal diameter of 0.2 mm [39] and tapered at the end so that the inner volume of the tapered tip would contain about 20 nl. Tapering was obtained by pulling the glass capillaries in a Micro-Pipette Puller (Industrial Science Associates, Inc., Ridgewood, NY). The embryo (or cell) was expelled directly onto the LysoDot in a volume of PBS as close as possible to 20 nl. Microscope observation revealed that the GITC crystals dissolved instantly upon addition of the sample-containing PBS and, thus, that cell lysis occurred immediately. Tubes were closed upside down and heated at 75–77°C for 5 minutes, after which their content was once again dry or semi-dry. The samples were then stored at -20°C until the next step.

In order to perform reverse transcription, each sample was carefully re-solubilized in the lid by addition of 6 μl of Random Hexamers mixture (4.2 ng/μl) in DEPC-treated water (all RT reagents were from a ThermoScript™ RT-PCR System kit, Invitrogen, Life Technologies, Carlsbad, CA). Tubes were closed, inverted, briefly centrifuged and incubated for 5 minutes at 65°C in order to allow primer/RNA hybridization. The remaining reagents needed for RT were then added to the tube in a volume of 4 μl, and the reaction was carried out according to the protocol suggested by the manufacturer. As previously described [17-19], all RT reagents were used at the suggested concentrations except for the absence of DTT, but volumes were halved so that each assay was performed in just 10 μl, which increased to 10.5 μl after RNase H digestion.

The full volume of each sample was then mixed with 89.5 μl of complete PCR amplification cocktail containing sequence-specific molecular beacons as detection probes [40]. Multiplex real-time PCR of *Xist *and *Sry *genomic DNA + cDNA templates was thus performed in a final volume of 100 μl, as detailed elsewhere, in the presence of 4 units of Taq DNA polymerase (Promega, Madison, WI) [18,19]. Real-time PCR was carried out in an ABI Prism^® ^7700 Sequence Detector (Applied Biosystems, Foster City, CA) and fluorescence readings were taken at the annealing temperature.

### PurAmp assay for *hsp70i *RNA and DNA measurements in individual embryos or blastomeres

Embryos were heat-shocked at 43°C for 30 minutes, followed by a recovery period of 30–40 minutes (blastocysts), or 2–3 hours (8-cell embryos, as indicated) at 37°C. The *hsp70i *assay was carried out similarly to the one for the *Xist/Sry *multiplex, by sequential dilutions of denaturant, RT and PCR reagents. The procedure for collection and lysis of the samples was the same. Dry samples were re-solubilized with 6 μl of random decamer primers (8.3 μM) in nuclease-free water (all RT reagents were from a Cells-to-cDNA™ II kit, Ambion, Inc., Austin, TX). After a 3 minute incubation at 75°C to optimize primer binding to RNA, all other reagents needed for RT were added to the sample and the reaction was carried out according to the manufacturer's instructions. As for the *Xist/Sry *assay, all RT mixture components were used at the suggested concentrations, but volumes were halved so that RT was performed in a final volume of 10 μl. An RNase H digestion step was included at the end of RT, as in the case of the *Xist/Sry *assay.

Real-time PCR was carried out in a final volume of 100 μl, by adding the PCR reagents to the sample after completion of RNase H digestion. The chosen *hsp70i *primers were localized at positions 1245/1305 of the *hsp70.1 *GenBank sequence with accession number M35021 (5' CCGCCTACTTCAACGAC 3', upstream primer; 5' ATCCGCAGCACGTTTA 3', downstream primer) and were identical to sequences within the *hsp70.3 *gene, previously known as *hsp70A1 *(GenBank sequence with accession number M76613) [14]. Because the *hsp70i *was the only amplicon generated in this assay, it was not necessary to design a sequence-specific detection probe. In this case, SYBR^® ^Green, a fluorescent dye that binds to double-stranded DNA, was used as fluorescent probe for real-time PCR. The specificity and purity of the amplicon was confirmed by both gel electrophoresis and analysis of the melt profile, as previously described [17]. The composition of the cocktail for *hsp70i *PCR was the following: 50 mM Tris, pH 8.3, 3 mM MgCl_2_, 0.3 μM each primer, 0.25 mM each dNTP, 1:62,500 SYBR Green (from a "10,000X concentrate in DMSO" purchased from FMC BioProducts, Rockland, ME), and 4 units of Taq DNA polymerase (Promega, Madison, WI). The polymerase was incubated at a 1:1 (v/v) ratio with Platinum^® ^Taq antibody (Invitrogen) for 5 minutes before addition to the reaction mixture (hotstart PCR). The cycling profile was: 95°C for 5 minutes; 10 cycles consisting of the following four steps: 95°C (20 sec), 64°C (30 sec), 72°C (30 sec), 84°C (15 sec); 35 cycles with the following four steps: 95°C (20 sec), 59°C (30 sec), 72°C (30 sec), 84°C (15 sec). Fluorescence readings were acquired at 84°C, in order to exclude fluorescent signals due to the possible formation of primer dimers late in the reaction.

A number of embryos were also processed as "No RT" controls, with the same protocol used for the other samples but without inclusion of reverse transcriptase in the RT mixture. These controls were used to quantify *hsp70i *genomic DNA copy numbers in the absence of cDNA [17].

### Multistep/multitube nucleic acid extraction

For the preliminary studies on *hsp70i *expression in blastocysts, some of the embryos were processed using a commercially available multistep/multitube kit, as previously described [18]. Briefly, nucleic acids (DNA and RNA) from each sample were purified using phenol:chloroform:isoamyl alcohol phase separation (Micro RNA Isolation Kit, Stratagene, La Jolla, CA) with a ratio of 100 μl of phenol and 45 μl of chloroform/isoamyl alcohol solution per assay. Transfer RNA (10 μg/assay; Sigma Chemical Company, St. Louis, MO) was added as a co-precipitant. Pellets were washed twice, once in isopropanol followed by overnight precipitation at -20°C and once in 75% ethanol, and then nucleic acids were reverse transcribed and analyzed by real-time PCR exactly as detailed above for the PurAmp-treated samples.

### Quantification of *Xist*, *Sry *and *hsp70i *amplicons

Calculation of template copy numbers was based on the "threshold cycle" (C_T_) at which each fluorescent signal was first detected above background. C_T _values were compared to standard scales obtained from analysis of male mouse genomes at known copy numbers, as detailed previously [17-19] and further exemplified in the Result section. Briefly, a two-fold difference in the number of templates amplified results in a shift of one cycle between two C_T _determinations. A lower C_T _value indicates an earlier detection of the fluorescent signal and therefore more templates present at the start of the reaction [reviewed in ref. [41]]. One male mouse genome contains one copy of *Sry *(Y-chromosome), one copy of *Xist *(X-chromosome) and four copies of *hsp70i *(two of *hsp70.1 *and two of *hsp70.3*, both on chromosome 17).

### Insertion of a DNase I digestion step within the *Xist/Sry *PurAmp protocol

Embryos were grown to the morula stage and were individually harvested and lysed as detailed above. Controls were processed for multiplex detection of *Xist/Sry *with the described PurAmp protocol. RNA-only samples were prepared by inserting a DNase digestion step prior to RT, as follows. Lysed, dried samples were re-suspended with 4 μl of DNase mixture containing: 20 mM Tris-HCl, pH 8.4; 2 mM MgCl_2_, 50 mM KCl (Invitrogen's DNase I Reaction Buffer) and 1 unit of DNase I (Ambion) in nuclease-free water. After an incubation of 20 minutes at room temperature, the reaction was terminated by adding 1 μl of a 10 mM EDTA solution, pH 8.0 (Invitrogen). The nuclease was inactivated by heating the samples at 65°C for 10 minutes, according to the protocol recommended by Invitrogen. One μl of a 25 ng/μl RT primer solution was then added to the sample, so that the final primer concentration was now the same as in the assay without DNase (see above). RT and PCR were then carried out as detailed for the "No DNase" assay.

## Authors' contributions

CH devised the finalized form of the PurAmp method, carried out the *Xist/Sry *measurements and drafted the manuscript. AA established heat shock conditions and performed the *hsp70i *quantification experiments. LJW coordinated the study and contributed to all aspects of its design.

## Figures and Tables

**Table 1:** Comparison of *hsp70i *copy numbers measured in single blastocysts: multistep/multitube versus PurAmp method

	***hsp70i *DNA + RNA copies**	
	Multistep/multitube extraction	PurAmp

No Heat shock, + RT	512 ± 69 (3)^a^	967 ± 548 (4)
Heat shock, + RT	2240 ± 1184 (3)	18,800 (2)

^a ^The number of single embryos averaged in each case is shown in parentheses.

**Table 2:** Correlation between *hsp70i *copy numbers in single blastomeres and whole 8-cell embryos, ± heat shock

	***hsp70i *DNA copies**	
No-heat shock, No RT Whole embryos (4)^a^ Per blastomere average ± s.d.	5 ± 2	

	***hsp70i *DNA + RNA copies**	

No-heat shock, + RT Whole embryos (4) Per blastomere average ± s.d.	19 ± 7	

Heat shock, + RT		
	Embryo 1^b^	Embryo 2^c^
Blastomere 1	459	750
Blastomere 2	311	705
Blastomere 3	438	281
Blastomere 4	667	730
Blastomere 5	563	491
Blastomere 6		1251
Per blastomere average ± s.d.	**488 ± 134**	**701 ± 325**
		
Whole embryos^b ^(6) Per blastomere average ± s.d	**500 ± 172**	
		
Whole embryos^c ^(2) Per blastomere average		**650**

^a ^The number of single embryos analyzed in each case is shown in parentheses.

^b ^Heat shock followed by 2-hour recovery prior to analysis.

^c ^Heat shock followed by 3-hour recovery prior to analysis.
